# Influenza

**Published:** 1889-12-28

**Authors:** 


### INFLUENZA.

As perhaps the majority of people will admit from personal experience, the symptoms of an ordinary coryza, catarrh, or "cold in the head," are much as follows: The individual attacked feels chilly, uncomfortable, with occasional flushes of heat, and probably headache. The nasal mucous membrane is at first dry, tumid, and irritable, and the uneasy sensations of which it is the seat prompt to coughing. The chest feels tight, stuffed, and constricted, and the eyes are suffused and watery. There is some hoarseness and soreness in the throat and windpipe, and irritation about the glottis, which again gives rise to cough. Sooner or later the nose runs, this often alternating with a stuffed feeling. Often with these symptoms, pains in the limbs like the pain of rheumatism occur. The appetite is impaired, the patient is thirsty, and general lassitude is felt all over the body. If the disorder pass down into the lungs chest symptoms increase, and frequently the term "cold in the chest" is applied to it; or from one of its prominent symptoms, "cough," or in medical language "bronchitis." In addition, the digestive organs are usually more or less affected, and there is furred tongue, loss of appetite, and thirst. Colds, as is well known, may lay the foundation of a number of ailments. Now the above symptoms are almost exactly the symptoms of influenza, which has, indeed, been called "epidemic catarrh." The symptoms of the influenza of 1833 were thus given by an eminent London physician. The patient is chilly and perhaps shivers, presently headache occurs and a sense of tightness across the forehead, the eyes become tender and watery, and sneezing and a copious defluxion from the nose ensue, followed or accompanied by heat and uneasiness about the throat, hoarseness, a troublesome cough, and oppression of breathing. With those symptoms a sudden and extraordinary subdual of strength and most commonly great depression of spirits. The tongue is white and creamy, the palate loses its sensibility, the appetite fails; nausea and vomiting are not uncommon. The patients complain of pain in the bowels and back. From the description we have had of the epidemic now prevailing, there can be no question that it is the same malady which occurred in this country in 1782, in 1803, in 1833, in 1837, and which has been designated *catarrhus e contagio*, the *grippe*, and by the Italians the influenza, or influence of the atmosphere. Admitting that this so-called influenza has prevailed in epidemic intensely in former years, we really cannot perceive any difference between it and a bad ordinary coryza and cold. It is said the febrile symptoms are more decided, that the cough is more troublesome, that the digestive organs are more implicated, that the depression and debility are more severe, that it is more likely to be followed by other affections, and that it kills more persons. Admitting all this, we do not regard it as a distinct disease. Very high fever sometimes attends an ordinary cold; cough is often decided enough; digestion may be altogether impeded; depression and debility could scarcely be more marked than they sometimes are; colds, as before observed, are the precursors of numerous other affections; and *catarrhus senilis*, or *catarrhus infantilis*, kills quite as many persons in proportion to those attacked as this so-called influenza. Now, influenza has been attributed to electricity, magnetic currents, spores of animal and vegetable origin floating in the atmosphere, pandemic waves, and a dozen other different agencies. But, as a matter of fact, we do not know what causes it any more than we know what causes ordinary coryza. The latter is generally attributed to chill, but there are not wanting observers who have opined that ordinary coryza is due to something more than chill, which may only be the exciting cause of an attack. It is perfectly well known that the malady called hay asthma may be excited by hay pollen floating in the atmosphere, or by the pollen from other vegetable growth even of roses, of vines, and of Roman wormwood. We do not pretend ability to define a reason for influenza, but we believe that when the cause of ordinary coryza has been discovered, the cause of influenza will be discovered. We believe that there are always cases of influenza in this country, which, when an epidemic of the kind is not prevalent, are regarded as coryza, catarrh, or bronchial irritation. It is unquestionable that all epidemic maladies have cycles of greater or lesser prevalence, under the operation of laws which we do not understand. The extreme probability is that this country will not escape an invasion of influenza; in fact, we have every reason to believe that it is now in London in greater force than usually the case. But we do not anticipate startling results. People, as a rule, are better housed, better fed, better clothed, and live under better sanitary conditions than they did even a few years back, and they are therefore better able to withstand the onslaught of influenza or any other epidemic disease. Avoiding chill is the great preventive means. Warmth, diaphoretics, expectorants, and quinine are the principal curative measures. And avoiding chill after an attack is the chief thing to attend to, in order to avoid unpleasant ulterior consequences.

